# Bridging the Polar and Hydrophobic Metabolome in Single-Run Untargeted Liquid Chromatography-Mass Spectrometry Dried Blood Spot Metabolomics for Clinical Purposes

**DOI:** 10.1021/acs.jproteome.1c00326

**Published:** 2021-07-23

**Authors:** Hanne Bendiksen Skogvold, Elise Mørk Sandås, Anja Østeby, Camilla Løkken, Helge Rootwelt, Per Ola Rønning, Steven Ray Wilson, Katja Benedikte Prestø Elgstøen

**Affiliations:** † National Unit for Screening and Diagnosis of Congenital Pediatric Metabolic Disorders, Department of Medical Biochemistry, Oslo University Hospital, Rikshospitalet, Sognsvannsveien 20, Oslo 0372, Norway; ‡ Department of Mechanical, Electronic and Chemical Engineering, Faculty of Technology, Art and Design, Oslo Metropolitan University, Pilestredet 35, Oslo 0166, Norway; § Department of Medical Biochemistry, Oslo University Hospital, Rikshospitalet, Sognsvannsveien 20, Oslo 0372, Norway; ∥ Department of Chemistry, University of Oslo, Sem Sælands vei 26, Oslo 0371, Norway; ⊥ Hybrid Technology Hub-Centre of Excellence, Institute of Basic Medical Sciences, Faculty of Medicine, University of Oslo, Domus Medica, Gaustad, Sognsvannsveien 9, Oslo 0372, Norway

**Keywords:** metabolomics, dried blood spots, LC−MS, inborn errors of metabolism

## Abstract

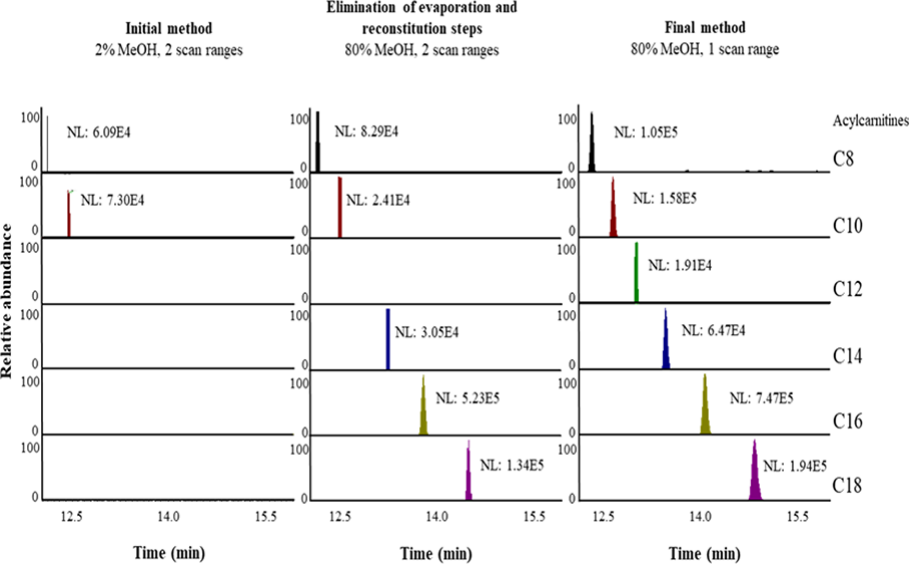

Dried blood spot (DBS) metabolite analysis is a central tool for the clinic, e.g., newborn screening. Instead of applying multiple analytical methods, a single liquid chromatography-mass spectrometry (LC–MS) method was developed for metabolites spanning from highly polar glucose to hydrophobic long-chain acylcarnitines. For liquid chromatography, a diphenyl column and a multi-linear solvent gradient operated at elevated flow rates allowed for an even-spread resolution of diverse metabolites. Injecting moderate volumes of DBS organic extracts directly, in contrast to evaporation and reconstitution, provided substantial increases in analyte recovery. Q Exactive MS settings were also tailored for sensitivity increases, and the method allowed for analyte retention time and peak area repeatabilities of 0.1–0.4 and 2–10%, respectively, for a wide polarity range of metabolites (log *P* −4.4 to 8.8). The method’s performance was suited for both untargeted analysis and targeted approaches evaluated in clinically relevant experiments.

## 1 Introduction

Reliable and accurate analyses of biological samples are essential for clinical purposes. Untargeted analyses of small biomolecules, i.e., metabolomics, provide the advantage of enabling the detection of large numbers of compounds at the same time. The approach has been described as having no discrimination in terms of which compounds can be detected within certain instrumental limitations, e.g., mass range.^1,2^ In human clinical samples, these metabolites include endogenous molecules, all the exogenous molecules that we are voluntarily and involuntarily exposed to, and a myriad of metabolites generated by our microbiomes.

Inborn errors of metabolism (IEMs) represent a large and diverse group of diseases.^3−5^ An IEM is typically caused by genetic mutations in a single gene, leading to changes in function or quantity of a vital enzyme. This in turn leads to deviations in the patient’s metabolism. IEMs can have serious consequences such as severe brain damage or death and are included in newborn screening programs in most countries. For IEM diagnostics, targeted analyses are mostly used, i.e., monitoring a limited number of predefined analytes. Untargeted analyses can also be of great importance for these diseases, as symptoms are often diverse and unspecific, making diagnostics based on targeted analyses difficult and potentially very time-consuming.^5^

Dried blood spots (DBS) are increasingly used in diagnostics due to ease of sampling, storage, and transportation, and higher stability of most analytes compared with, e.g., plasma or whole blood samples.^6−8^ Palmer et al. reported detecting more metabolites in DBS than in plasma samples, likely due to red and white blood cell metabolites.^9^ DBS are also widely used in newborn screening,^10,11^ allowing newborn screening even in remote areas, as samples can be gathered anywhere in the world and sent to laboratories elsewhere for analysis.

Mass spectrometry (MS) is a key tool in metabolomics.^12−14^ MS enables reliable and accurate identification of compounds and generally provides increased sensitivity compared to, e.g., NMR spectroscopy techniques.^15^ Regarding clinical metabolic screening, methods may be built upon direct infusion mass spectrometry due to, e.g., speed and simplicity.^10,16^ On the other hand, applying liquid chromatography (LC) upstream to MS has the advantage of providing additional separation of compounds, thereby increasing the number of identifiable compounds and reduction of matrix effects such as ion suppression and ion enhancement.^14,17^ Applying LC–MS also increases the chance of identification of isomers and provides the additional parameter of retention time for identification purposes.^14,18^

A key challenge with analyses of biological samples is the wide polarity range of clinically relevant metabolites, ranging from, e.g., hydrophobic fatty acids to polar amino acids, often addressed by using several analytical methods/platforms, e.g., employing gas chromatography in addition to hydrophilic interaction liquid chromatography (HILIC) and reversed phase LC, to cover a broad part of the metabolome.^19−23^ Although comprehensive analysis is provided, multi-separation approaches can be time-consuming and laborious, sometimes requiring extensive sample preparation procedures. Several single metabolomics methods have been developed for, e.g., plasma and urine analysis, with excellent results (see, e.g., refs (24) and (25)). Broad range single methods are even beginning to merge different omics; He et al. have recently combined lipidomics and proteomics with a single-shot technology.^26^ We set out to develop and optimize a single method suited for covering a broad range of metabolites focusing on dried blood spots while still ensuring simplicity for practical clinical use, e.g., IEM diagnostics. We were interested if a single LC–MS method would have satisfactory analytical performance (for example, robust retention times and peak area measurements) for metabolites ranging from sugars to lipids.

In this work, we present optimizations and demonstrations of a metabolomics LC-Orbitrap MS method for DBS analysis intended to “bridge” the hydrophobic and polar metabolome in a single run. We focus on factors including retention time/peak area stability, selectivity/peak capacity, DBS extraction, and MS parameter settings. We demonstrate the method for both untargeted analysis and a targeted approach.

## 2 Materials and Methods

### 2.1 Equipment

Filter paper cards used were Whatman 903 Protein Saver cards (GE Healthcare Life Sciences, Chicago, IL, USA). A manual puncher from McGill (Advantus Corp., Jacksonville, FL, USA) was used to punch the DBS. Microtubes were obtained from Sarstedt (Nümbrecht, Germany). For extraction, a Thermomixer Comfort (Eppendorf, Hamburg, Germany) was used. Glass tubes for evaporation to dryness were obtained from VWR (Radnor, PA, USA), and the evaporator used was a TurboVap LV (Caliper Life Sciences, Waltham, MA, USA). HPLC vials, caps, and inserts were from La-Pha-pack (Thermo Scientific, Waltham, MA, USA).

The following LC columns were evaluated: Polaris C18-Ether and Pursuit XRs Diphenyl (both from Agilent Technologies (Santa Clara, CA, USA)), C18-Pentafluorophenyl (PFP) from ACE Technologies (Aberdeen, Scotland), Aeris Peptide XB-C18 (Phenomenex (Torrance, CA, USA)), and Raptor Biphenyl (Restek (Bellefonte, PA, USA)). For column specifications, see Table 1.

**Table 1:** Specifications of Evaluated Columns

column	length, mm	diameter, mm	particle size, μm	pore size, Å	surface area, m^2^/g	carbon load, %
Polaris C18-Ether	250	2.0	3.0	180	200	12
Pursuit XRs Diphenyl	250	2.0	3.0	100	440	15
ACE C18-PFP	250	2.1	3.0	100	300	14
Aeris Peptide XB-C18	250	2.1	3.6	100	200	10
Raptor Biphenyl	150	2.1	2.7	90	150	7

### 2.2 Chemicals and Solvents

All water used was of type 1 (>18 MΩ cm), obtained from a MilliQ ultrapure water purification system (Merck Millipore, Darmstadt, Germany). Methanol was obtained from Rathburn Chemicals (Walkerburn, Scotland). Formic acid (98%) was obtained from Merck.

Tobramycin, acylcarnitines C2, C12, and C16, D2 glycolic acid, D6 glucose, and acylcarnitines D3 C2, D3 C12, and D3 C16 were obtained from Larodan (Solna, Sweden). D4 succinic acid, ^13^C creatine, and uric acid were purchased from Sigma (Darmstadt, Germany). Vancomycin (1000 mg powder) was obtained from MIP Pharma GmbH (Blieskastel, Germany). ^13^C_2_ guanidinoacetate was obtained from Dr. H Ten Brink (VU University Medical Center, Amsterdam, The Netherlands). Creatinine was obtained from Merck. Creatine was obtained from Nutritional Biochemical Corporation (Cleveland, OH, USA).

### 2.3 Method Development

The following parameters were evaluated and optimized: MS parameters, with regard to signal intensity: electrospray voltage (evaluated values: 1, 2, 3.5, 4, 5, 6, and 7 kV), electrospray needle position (evaluated positions: A–D, A being the closest to the inlet), resolution (evaluated values: 17500, 35,000, 70,000, and 140,000 FWHM (at *m*/*z* 200)), and automatic gain control (AGC) target value (evaluated values: 2E4, 5E4, 1E5, 2E5, 5E5, 1E6, 3E6, and 5E6 ion counts). The evaluation of using either a broad (and split) scan range of *m*/*z* 50–750 and 750–1700 (performed with one analysis) or only one range of *m*/*z* 50–750 was done with regard to peak area.

**Figure 1:**
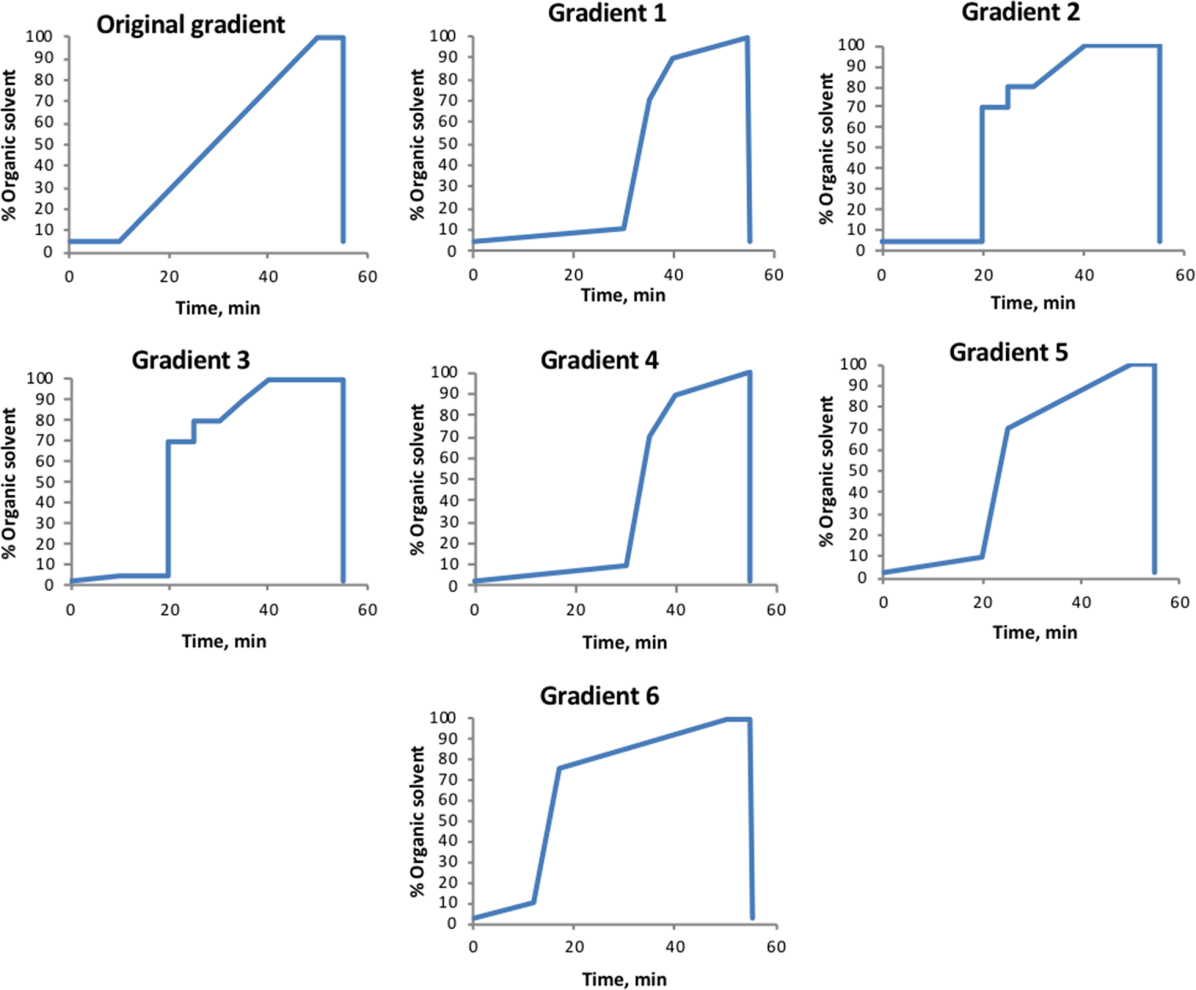
Gradient profiles tested. Mobile phase A: water with 0.1% formic acid. Mobile phase B: methanol with 0.1% formic acid.

LC parameters, with regard to peak distribution, are as follows: LC column (see Table 1 for names and specifications) and gradient elution profile (see Figure 1 for evaluated profiles; more details are described below). LC parameters, with regard to peak capacity, are as follows: injection volume (evaluated volumes: 2, 10, and 20 μL) and mobile phase flow rate (evaluated rates: 150 and 300 μL/min).

Figure 1 shows the evaluated gradient elution profiles (evaluated using a Pursuit XRs Diphenyl column). The original gradient was the starting point of the gradient profile optimization, while gradients 1–6 were defined based on when compounds in a spiked DBS eluted. Total analysis time was later reduced to 32.5 min.

Sample preparation optimization is as follows: evaluation of evaporating samples to dryness and re-solving in 2, 40, and 80% methanol, respectively, versus injection of an 80% methanol extract without evaporation to dryness, with regard to peak area. DBS punch location (evaluated locations: A–D, A being in the center of the spot and D in the perimeter (see Figure 6 in the Results and Discussion section)), with regard to standard deviation of the measured peak area (10 spots were used, providing 10 punches from each location) was evaluated.

### 2.4 Sample Preparation

For optimization experiments, whole blood from a healthy volunteer was used. The whole blood sample was either spotted onto a filter paper card directly or mixed with aqueous standards (50:50 v/v) and spotted onto a filter paper card. The same blood sample was used in all experiments for optimization of a chosen parameter. The whole blood sample was either mixed with the standards directly before spotting or stored in a freezer at −80 °C and thawed before mixing and spotting.

Dried blood spots were either made immediately prior to each experiment, or prepared samples were stored at −80 °C before use. The following steps constitute the final sample preparation: 3.2 mm punches were punched from DBS (∼3 μL of whole blood or ∼1.5 μL of whole blood for the samples consisting of whole blood mixed with aqueous standards) and extracted in a microtube with 100 μL of 80% aqueous methanol with 0.1% formic acid using a thermomixer for 45 min (at 45 °C, 700 rpm). Samples were transferred to an HPLC vial for analysis directly after extraction.

### 2.5 Instrumentation

LC instrumentation used was a Dionex Ultimate 3000 UHPLC quaternary system pump, column department, and autosampler, from Thermo Scientific. The MS used was a Q Exactive Orbitrap (Thermo Scientific). The ionization source was an electrospray, and samples were analyzed in both positive and negative modes (in separate injections).

### 2.6 Settings and Details

The following settings constitute the final method. The LC column used was a Pursuit XRs Diphenyl (see Table 1 for details). The injection volume was 2 μL. The mobile phase (A: water with 0.1% formic acid and B: methanol with 0.1% formic acid) flow rate was 300 μL/min. The gradient elution profile was profile 6 in Figure 1. Column temperature was 30 °C, and total analysis time was 32.5 min. Re-equilibration time was 10 min.

The scan type was full MS (scan range *m*/*z* 50–750). The resolution was 70,000 FWHM (at *m*/*z* 200). The AGC target value was 1,000,000 ion counts. Maximum injection time was 250 ms. Electrospray settings are the following: sheath gas (N_2_) flow rate: 40 (a.u.), auxiliary gas (N_2_) flow rate: 10 (a.u.), sweep gas (N_2_) flow rate: 2 (a.u.), capillary temperature: 250 °C, S-lens RF level: 50, auxiliary gas heater temperature: 300 °C, electrospray voltage: 3.5 kV, and electrospray needle position: C.

Equation 1 was used for calculation of peak capacity (*P*_c_)
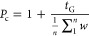
1

where *t*_G_ is the gradient elution time, *n* is the number of peaks, and *w* is the peak width at the baseline (13.4% peak height) for each peak.

### 2.7 Computer Software

Software used was Xcalibur (Version 4.2.47), Tune (version 2.11), and SII for Xcalibur 1.5, all from Thermo Scientific. Compound Discoverer 2.1 (Thermo Scientific) was used for data processing.

### 2.8 Approval by the Regional Committee for Medical and Health Research Ethics

The use of whole blood from healthy volunteers was approved by the Regional Committee for Medical and Health Research Ethics (case no.: 173346).

## 3 Results and Discussion

An LC-Orbitrap MS method for metabolomics analyses of DBS was optimized regarding sample preparation, chromatographic properties, and MS conditions for coverage of a broad range of metabolites and a high degree of sensitivity. A selection of endogenous and isotopically labeled metabolites (spiked in controlled amounts) was used for the optimization experiments. Included in this list were hydrophobic acylcarnitines (key biomarkers in newborn screening) as well as more polar amino acids including valine and tyrosine (biomarkers of maple syrup urine disease and tyrosinemia, respectively). Using the same methods, proof-of-concept demonstrations were performed for both targeted and untargeted DBS applications. Below is a more detailed presentation of the optimizations, method evaluation, and proof-of-concept experiments.

### 3.1 Method Development

#### 3.1.1 MS Optimization

**Figure 2:**
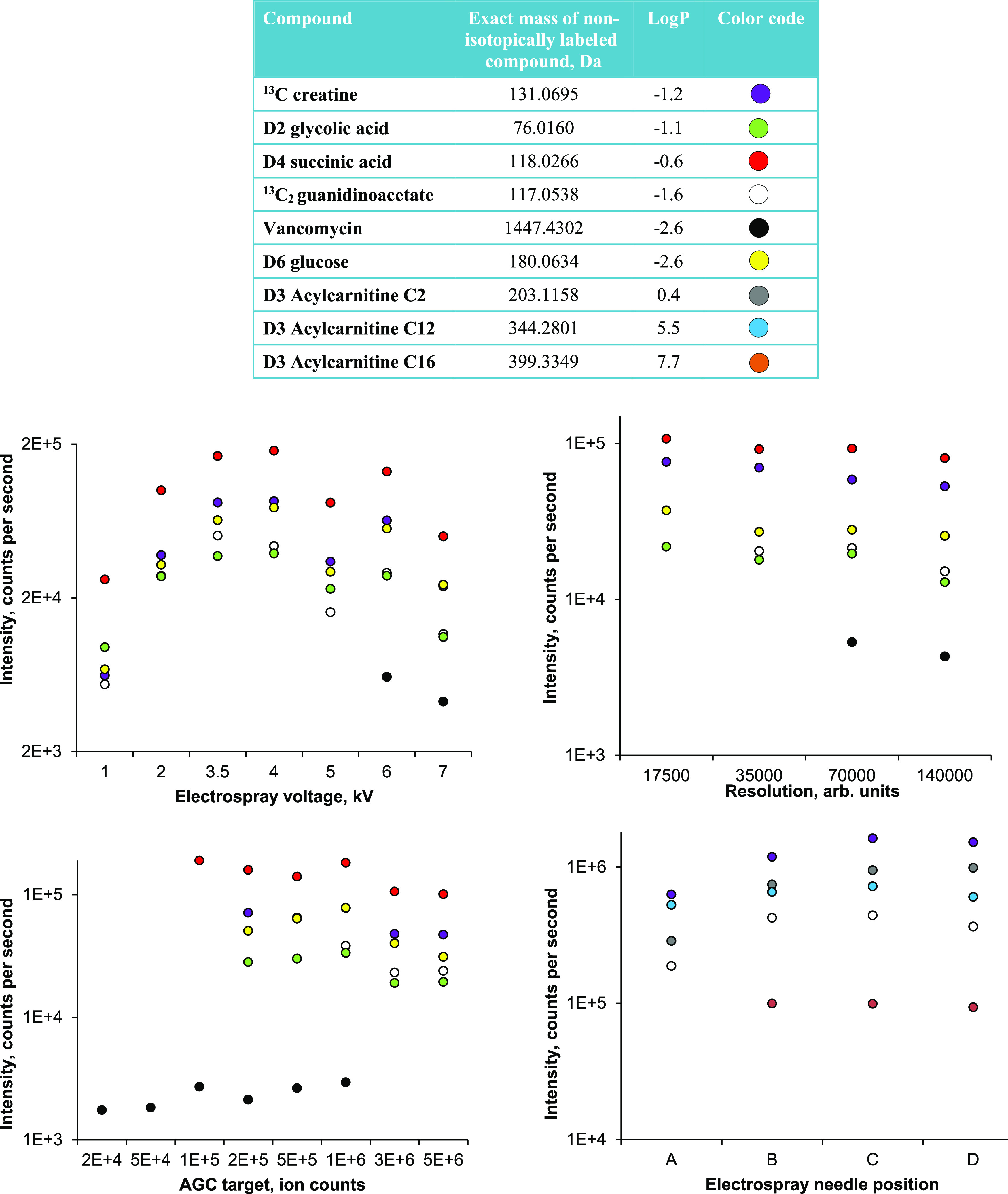
Top: exact mass and log *P* values of compounds used for MS optimization experiments. Exact mass and log *P* values were found at Pubchem.^27^ Colored dots show the color of each compound in the plots. Bottom: peak intensity with tested settings.

Q Exactive mass spectrometry parameters were optimized for metabolites from DBS with regard to signal intensity. For optimization of MS parameters, we used a standard mix of metabolites (about 5 μmol/L each) with a range of molecular weights, structures, and polarities (see Figure 2). In these experiments, all monitored standards were isotopically labeled to ensure that observed changes in signal intensities were caused only by parameter settings without interference from endogenous contributions (except for the drug vancomycin). Vancomycin was added to include a compound with a relatively large mass that is also analyzed in our routine laboratory, making method comparison possible. Optimization experiments were primarily performed using an aqueous mix of standards mixed with whole blood (50:50 v/v) and spotted onto a filter paper card. For these samples, the following MS settings were considered to be the best choices: electrospray voltage of 3.5 kV, electrospray needle position C (options were A–D, A being the closest to the inlet, with a difference between the positions of approximately 3.5 mm), resolution of 70,000 FWHM (at *m*/*z* 200), and automatic gain control (AGC) target value of 1,000,000 ion counts. See Figure 2 for comparison to other settings. AGC target values and electrospray voltage were of highest significance regarding sensitivity. Importantly, sensitivity increased significantly (92–109% increase in the peak area of investigated compounds) when changing from a split scan range (*m*/*z* 50–750 and 750–1700) to one scan range (*m*/*z* 50–750).

#### 3.1.2 LC Optimization

**Figure 3:**
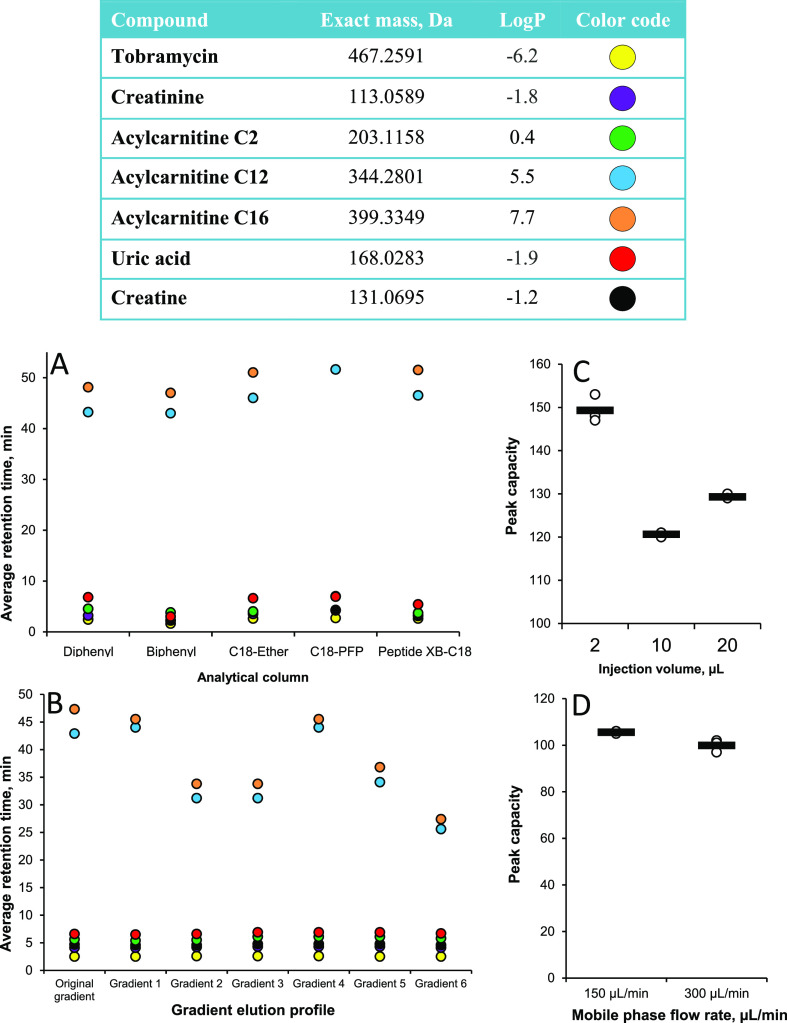
Top: exact mass and log *P* values of compounds used for LC optimization experiments. Exact mass and log *P* values were found at Pubchem.^27^ Colored dots show the color of each compound in the plots. Bottom: average retention time (*n* = 3) of compounds for tested columns (A). All columns tested provided sharp chromatographic peaks (see the Supporting Information, Figures S1–S4), but the diphenyl SP variant was interpreted as having a modestly best selectivity for polar compounds (0–10 min area). (B) Retention times for gradient elution profiles using the diphenyl SP, with gradient 6 arguably preserving selectivity at a shorter analysis time. Average peak capacity (*n* = 3) for tested injection volumes (C) and tested mobile phase flow rates (D).

Chromatographic parameters were optimized for metabolites from DBS first with regard to selectivity (here focusing on the ability to separate neighboring polar compounds while separating neighboring nonpolar compounds from each other) and subsequently peak capacity. In these experiments, the monitored standards were non-isotopically labeled metabolites but related to those in the MS experiments (see Figure 3). Optimization experiments were primarily performed using an aqueous mix of standards mixed with whole blood (50:50 v/v) and spotted onto a filter paper card. The following LC parameter settings were considered to be the best choices (see Figure 3 for comparison to other settings): analytical column: Pursuit XRs Diphenyl (hydrophobic + π–π interactions provided a modest increased selectivity of the model polar analytes), multi-linear gradient elution profile 6 in Figure 1 (interpreted as the best in maintaining the diphenyl column’s selectivity within a shortened analysis time), injection volume of 2 μL (associated with the highest peak capacity when using gradient profile 6, e.g., due to not overloading the column), and mobile phase flow rate of 300 μL/min (no large difference in peak capacity between tested flow rates, allowing a further reduction of the analysis time).

#### 3.1.3 Sample Preparation Optimization

**Figure 4:**
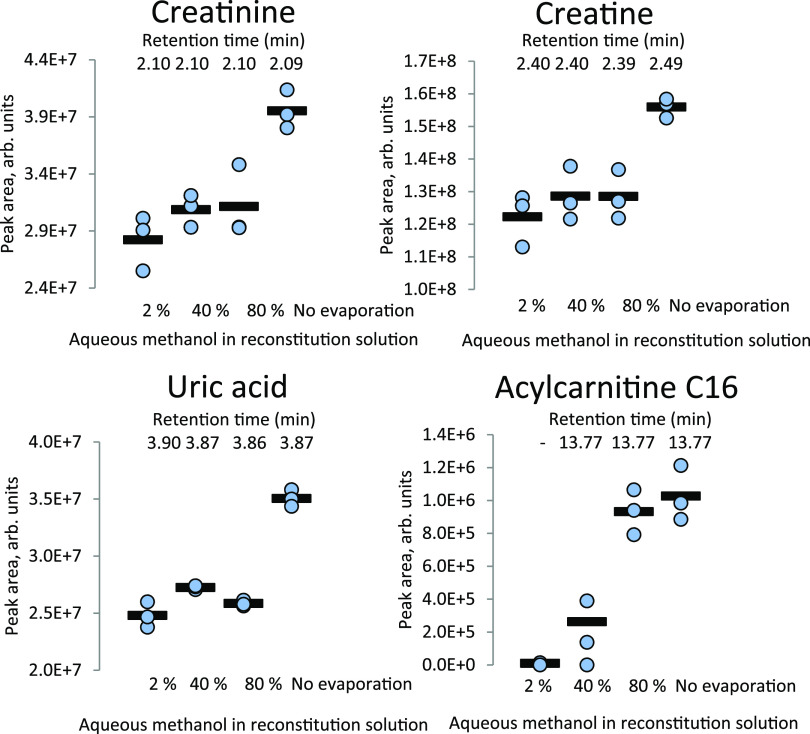
Improved sensitivity with an increased amount of methanol. Peak area in DBS samples prepared with 2, 40, and 80% aqueous methanol in the reconstitution solution and with the exclusion of the evaporation and reconstitution steps. Retention times of the compounds for each organic solvent concentration are shown.

Sample preparation parameters were optimized for metabolites from DBS with regard to recovery/detection capability. The following steps and settings constitute the final sample preparation protocol: extraction of one punch from a DBS with 100 μL of 80% aqueous methanol with 0.1% formic acid and thermo-mixing for 45 min at 45 °C (700 rpm). The solution was directly transferred to an HPLC vial, as this gave improved recovery and detection (28–40% increase) for all compounds tested compared to evaporating to dryness and re-solving in the starting mobile phase (Figure 4). Although a centrifugation step was not included, we did not experience blockages of the LC column. The simplified procedure of injecting higher organic contents did not lead to substantial changes in retention time for any of the compounds investigated.

**Figure 5:**
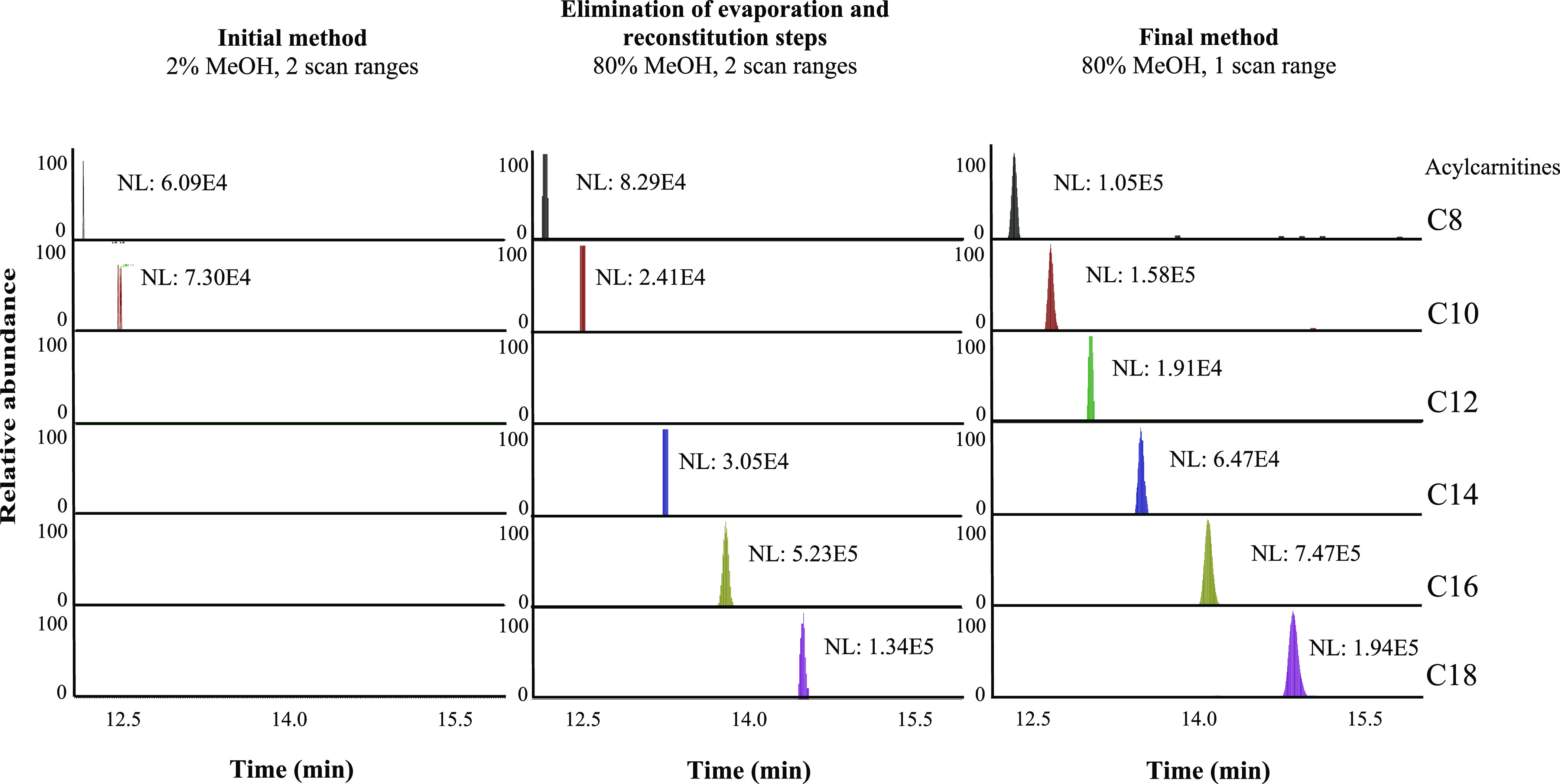
Extracted ion chromatograms of a selection of acylcarnitines showing detection improvement when changing from evaporation to dryness and a broad and split scan range (*m*/*z* 50–750 and 750–1700) to direct transfer of the extract to an HPLC vial and a narrow scan range (*m*/*z* 50–750).

As shown in Figure 5, eliminating the evaporation to dryness step and changing from a broad and split (*m*/*z* 50–750 and 750–1700) to a narrow (*m*/*z* 50–750) scan range significantly improved detection of acylcarnitines.

**Figure 6:**
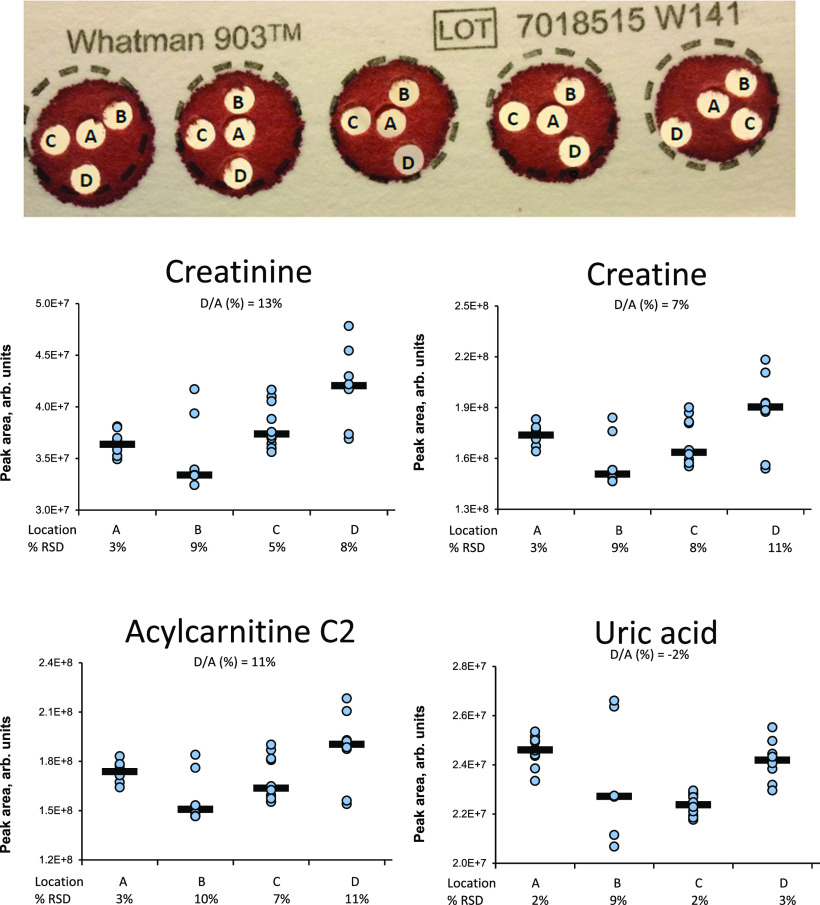
Top: punch locations tested. Ten spots were used, providing 10 punches from each location. Bottom: peak areas with RSD % of selected compounds in DBS samples prepared with punches taken from locations A, B, C, and D.

To evaluate potential differences in punch location within the DBS, four punch locations were investigated (see Figure 6). We observed a larger relative standard deviation (RSD %) in punches taken from the perimeter of the spot compared to the center. Center punches were thus considered to be the best choice.

### 3.2 Evaluation of Peak Area Linearity

**Figure 7:**
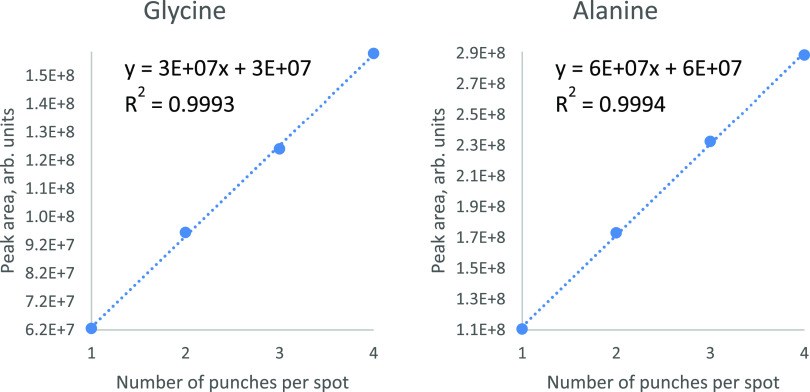
Linear increase in peak area with an increasing number of punches from one spot for glycine and alanine.

To evaluate the abilities of our DBS MS platform, we investigated the effect on the measured peak area of endogenous metabolites when increasing the number of DBS punches (1, 2, 3, and 4 punches, equivalent to about 3, 6, 9, and 12 μL of whole blood, respectively). Peak area linearity was overall satisfactory for the investigated analytes. As shown in Figure 7, with glycine and alanine as examples, peak area increased linearly with an increasing number of punches (*R*^2^ ranging from 0.9358 = ornithine to 0.9994 = alanine). To monitor instrument performance and repeatability, the same DBS sample (healthy volunteer) was injected three times each day during an analysis run of 11 days. Table S1 (Supporting Information) shows a high repeatability regarding average retention time and peak area (0.1–0.4 and 2–10%, respectively).

### 3.3 Application/Proof of Concept

#### 3.3.1 Targeted Approach

**Figure 8:**
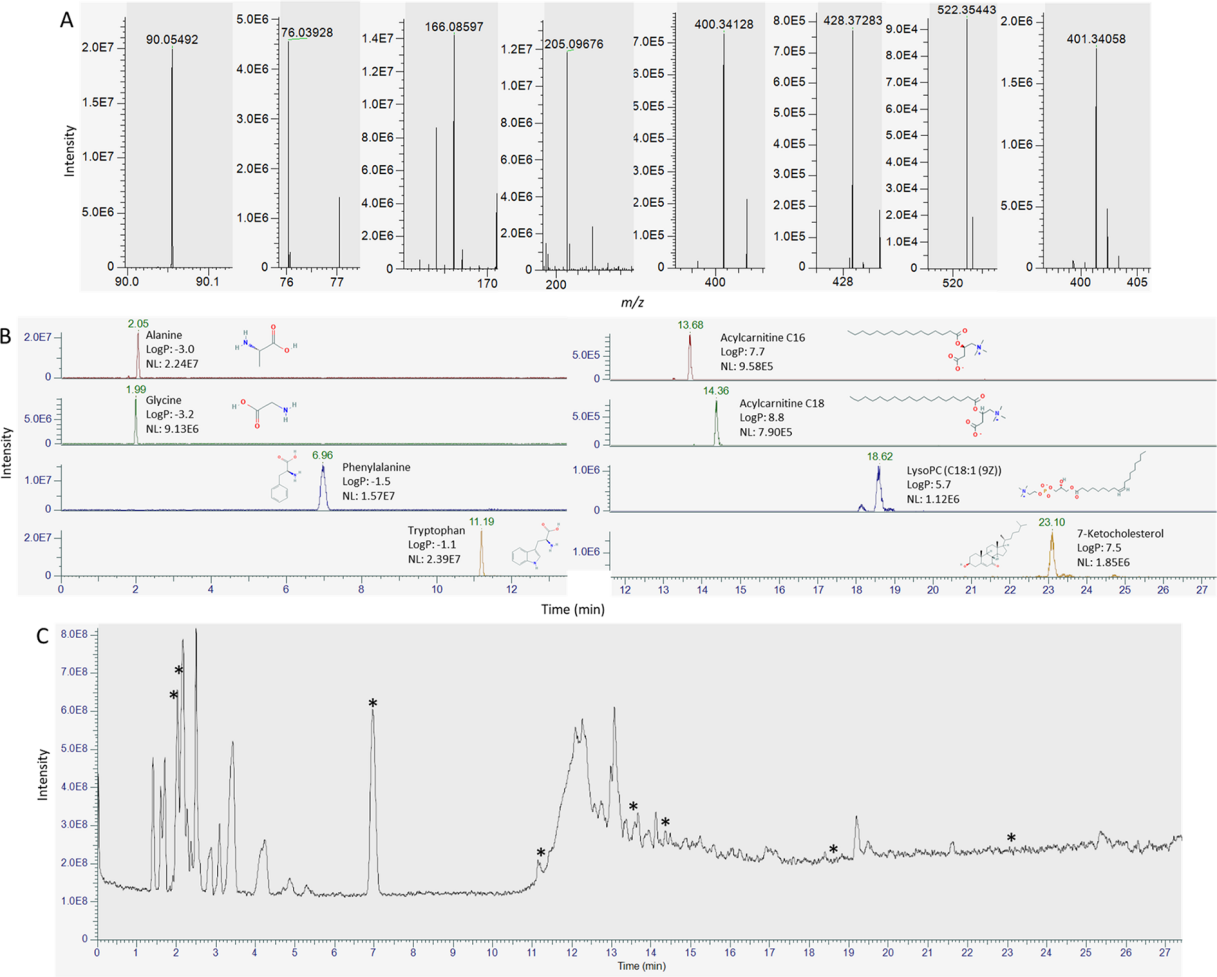
Mass spectra (A) and extracted ion chromatograms with names, log *P* values, signal intensities, and structures (B) of a selection of detected endogenous compounds in a dried blood spot (alanine, glycine, phenylalanine, tryptophan, acylcarnitine C16, acylcarnitine C18, LysoPC (C18:1 (9Z)), and 7-ketocholesterol), and total ion chromatogram (C) with asterisks indicating at which points in the chromatogram the spectra in panel (A) are located. Log *P* values and structures were obtained from Pubchem.^27^

The method covers a large part of the metabolome. Endogenous metabolites ranging in polarity from log *P* −4.4 to 8.8 are readily detected; see Table 2 for a list of representative compounds, highlighted here as they are all employed as biomarkers in, e.g., newborn screening and for maple syrup urine disease and tyrosinemia (among other diseases). In addition, Figure 8 shows a total ion chromatogram from a positive ionization dried blood spot analysis with mass spectra and extracted ion chromatograms of eight selected detected endogenous compounds, illustrating the even peak distribution of hydrophilic and hydrophobic compounds along the chromatogram.

**Table 2:** Examples of Detected Endogenous Compounds with a Wide Range of Polarities^a^

amino acid	chemical formula	exact mass [M + H]^+^, Da	accurate mass [M + H]^+^, Da	mass error, ppm	log *P*	acylcarnitine	chemical formula	exact mass, [M + H]^+^, Da	accurate mass [M + H]^+^, Da	mass error, ppm	log *P*
ornithine	C_5_H_12_N_2_O_2_	133.0972	133.0970	–1.50	–4.4	C0	C_7_H_15_NO_3_	162.1125	162.1122	–1.85	–0.2
citrulline	C_6_H_13_N_3_O_3_	176.1030	176.1027	–1.70	–4.3	C2	C_9_H_17_NO_4_	204.1230	204.1227	–1.47	0.4
arginine	C_6_H_14_N_4_O_2_	175.1190	175.1187	–1.71	–4.2	C3	C_10_H_19_NO_4_	218.1387	218.1383	–1.83	0.9
glycine	C_2_H_5_NO_2_	76.0393	76.0393	0.00	–3.2	C4	C_11_H_21_NO_4_	232.1543	232.1539	–1.72	1.2
alanine	C_3_H_7_NO_2_	90.0550	90.0549	–1.11	–3.0	C5	C_12_H_23_NO_4_	246.1700	246.1695	–2.03	1.8
valine	C_5_H_11_NO_2_	118.0863	118.0861	–1.69	–2.3	C8	C_15_H_29_NO_4_	288.2169	288.2162	–2.43	3.4
tyrosine	C_9_H_11_NO_3_	182.0812	182.0810	–1.10	–2.3	C12	C_19_H_37_NO_4_	344.2795	344.2782	–3.78	5.5
methionine	C_5_H_11_NO_2_S	150.0583	150.0581	–1.33	–1.9	C14	C_21_H_41_NO_4_	372.3108	372.3103	–1.34	6.6
leucine	C_6_H_13_NO_2_	132.1019	132.1017	–1.51	–1.5	C16	C_23_H_45_NO_4_	400.3421	400.3414	–1.75	7.7
phenylalanine	C_9_H_11_NO_2_	166.0863	166.0860	–1.81	–1.5	C18	C_25_H_49_NO_4_	428.3734	428.3728	–1.40	8.8

a Log *P* values were obtained from Pubchem.^27^

**Figure 9:**
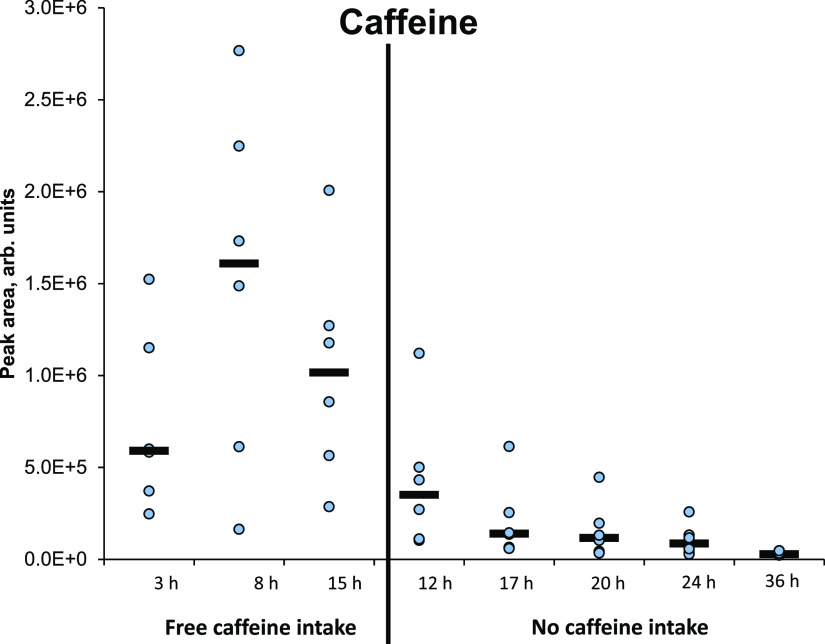
Measured amounts of caffeine decreased with time since intake. The spread in the measured peak area is likely caused by different intakes of coffee between the participants.

We also investigated if our method could detect changes in the concentration of one target metabolite out of the thousands of metabolites detected. Six healthy volunteers were taken DBS samples during free intake of coffee and soda for 15 h (during which the participants consumed one to four cups of coffee each) and during no intake of coffee and soda (with the first sample taken after 12 h of no caffeine intake). Caffeine is a suitable compound to monitor as we know that it is exogenous (mostly originating from coffee and soda), and people consume various amounts of the substance. Thus, caffeine was measured in all samples. As shown in Figure 9, the measured amount of caffeine decreased with increasing time since intake.

#### 3.3.2 Detection of Differences in Nutritional States (Untargeted Analysis)

**Figure 10:**
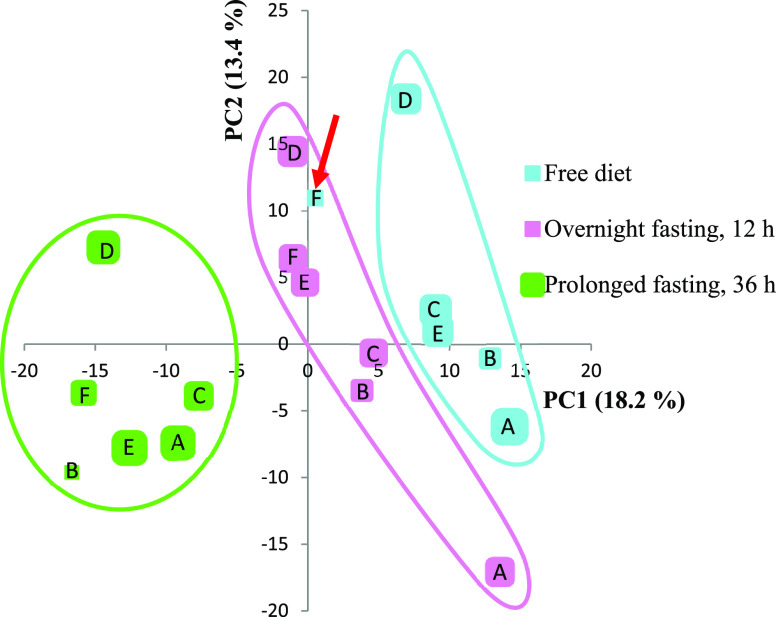
Principal component analysis plot of DBS samples from six individuals (A–F) taken after free diet, overnight fasting (12 h), and prolonged fasting (36 h). One point represents metabolites detected in that particular sample. Individual F did not eat before collecting the free diet sample. This point (red arrow) thus clustered correctly together with the overnight fasting samples.

An additional proof of concept study was performed by analyzing DBS samples from six healthy volunteers during free food intake and during fasting. DBS samples were taken during free diet, after 12 h of fasting, and after 36 h of fasting. The volunteers were allowed to drink as much water as they wanted during the fasting period. A principal component analysis plot of samples taken from all volunteers during free diet and after 12 h and 36 h of fasting is shown in Figure 10. The samples from the three nutritional states clearly grouped as three separate clusters, with the apparent exception of the free diet sample from person F, which clustered together with samples taken after 12 h of overnight fasting. However, it turned out that person F was actually omitting breakfast that day, meaning that this point is correctly located together with the overnight fasting samples and should in fact be classified as an overnight fasting sample.

**Figure 11:**
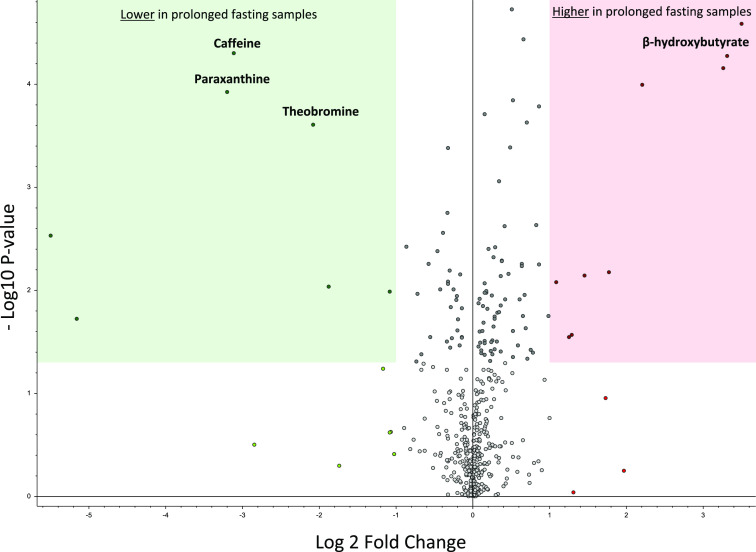
Volcano plot of DBS samples from six individuals taken after overnight fasting (12 h) and prolonged fasting (36 h). One point represents one compound. Green box: lower concentration measured in samples taken after 36 h than after 12 h of fasting. Red box: higher concentration measured in samples taken after 36 h than after 12 h of fasting.

To evaluate the method’s ability to identify discriminating compounds between groups, we used a volcano plot to compare samples taken after overnight fasting (12 h) with samples taken after prolonged fasting (36 h) (Figure 11). Compounds with a significantly lower concentration in prolonged fasting samples were identified: caffeine, theobromine, and paraxanthine, all associated with metabolism of (coffee) drinks.^28,29^ An upregulated marker of prolonged fasting samples was identified as β-hydroxybutyrate, a ketone body naturally produced during fasting for energy transfer.^30^ Taken together, the single run platform was well suited for revealing trends of the expected metabolism in this controlled study.

## 4 Conclusions

A single LC–MS method has satisfactory analytical performance for a broad range of metabolites with regard to polarity, having evaluated and optimized parameters regarding MS sensitivity, column separation, and sample preparation, demonstrated with proof-of-concept studies regarding both untargeted and targeted metabolite approaches using the same method/settings. We find that a single LC–MS method can indeed be a compromise between multi-method deep profiling and fast “shotgun” approaches. We have here evaluated our platform with regard to key biomarkers of inborn errors of metabolism and are currently exploring the limitations of our platform regarding lipids such as phosphatidylcholines, cholesterol esters, and acylglycerols (employing SPLASH standards), improving the identification abilities of our platform with the use of about 600 metabolite standards (MSMLS by IROA Technologies), and exploring the quantitative abilities of our platform by comparing with standard clinical chemistry.

## Supporting Information Available

(Table S1) Relative standard deviations of retention time and peak area of selected compounds measured in a DBS, number of detected features and annotated/identified metabolites, using Compound Discoverer 2.1, in a dried blood spot positive ionization analysis, and (Figures S1–S4) chromatograms from other evaluated columns (PDF)
