# Supplementary material for: Bridging the Polar and Hydrophobic Metabolome in Single-Run Untargeted Liquid Chromatography-Mass Spectrometry Dried Blood Spot Metabolomics for Clinical Purposes
Source: J Proteome Res. 2021 Jul 23;20(8):4010–21. doi: 10.1021/acs.jproteome.1c00326 (PMC8397434; doi:10.1021/acs.jproteome.1c00326)
Supplement: Supplementary file 1 — pr1c00326_si_001.pdf [file pr1c00326_si_001.pdf]

Supplementary file for:

Bridging the polar and hydrophobic metabolome in single-run untargeted liquid chromatography-mass spectrometry dried blood spot metabolomics for clinical purposes

Hanne Bendiksen Skogvold[1, 2], Elise Mørk Sandås[1], Anja Østeby[1], Camilla Løkken[1], Helge Rootwelt[3], Per Ola Rønning[2], Steven Ray Wilson[4,5], Katja Benedikte Prestø Elgstøen[1*]

[1] National Unit for Screening and Diagnosis of Congenital Pediatric Metabolic Disorders, Department of Medical Biochemistry, Oslo University Hospital, Rikshospitalet, Sognsvannsveien 20, 0372 Oslo, Norway

[2] Department of Mechanical, Electronic and Chemical Engineering, Faculty of Technology, Art and Design, Oslo Metropolitan University, Pilestredet 35, 0166 Oslo, Norway

[3] Department of Medical Biochemistry, Oslo University Hospital, Rikshospitalet, Sognsvannsveien 20, 0372 Oslo, Norway

[4] Department of Chemistry, University of Oslo, Sem Sælands vei 26, 0371 Oslo, Norway

[5] Hybrid Technology Hub-Centre of Excellence, Institute of Basic Medical Sciences, Faculty of Medicine, University of Oslo, Domus Medica, Gaustad, Sognsvannsveien 9, 0372 Oslo, Norway

Corresponding author:

Katja Benedikte Prestø Elgstøen (kelgstoe@ous-hf.no)

Supplementary Table

Table S1. Peak area (PA) and retention time (RT) relative standard deviation (RSD %) of selected compounds measured in a DBS injected three times each day during an analysis run of 11 days.

Compound	Average PA (a.u.) N=30	RSD % PA (a.u.) N=30	Average RT (min) N=30	RSD % RT (min) N=30
**Ornithine**	1.81E+07	4 %	1.62	0.3 %
**Arginine**	6.88E+06	3 %	1.75	0.4 %
**Citrulline**	1.75E+07	3 %	2.19	0.2 %
**Valine**	1.71E+08	9 %	2.50	0.1 %
**Methionine**	8.17E+06	7 %	3.05	0.2 %
**Leucine**	1.37E+08	6 %	3.33	0.3 %
**Tyrosine**	3.34E+07	5 %	4.10	0.3 %
**Phenylalanine**	6.73E+07	3 %	6.73	0.2 %
**Acylcarnitine C0**	5.16E+07	2 %	2.11	0.3 %
**Acylcarnitine C2**	3.10E+07	4 %	2.61	0.2 %
**Acylcarnitine C3**	1.02E+06	8 %	4.97	0.4 %
**Acylcarnitine C8**	2.09E+05	8 %	12.09	0.1 %
**Acylcarnitine C14**	3.35E+05	10 %	13.14	0.1 %
**Acylcarnitine C16**	2.90E+06	4 %	13.69	0.1 %
**Acylcarnitine C18**	1.14E+06	4 %	14.42	0.1 %

Supplementary Information

Number of detected features and annotated/identified metabolites

An example of the number of detected features and annotated/identified metabolites, using Compound Discoverer 2.1 (with standard settings and filters for a metabolomics workflow), in a dried blood spot positive ionization analysis: detected features: 11394. Annotated or identified metabolites: 1042.

Supplementary figures S1-S4 (chromatograms from other evaluated columns)

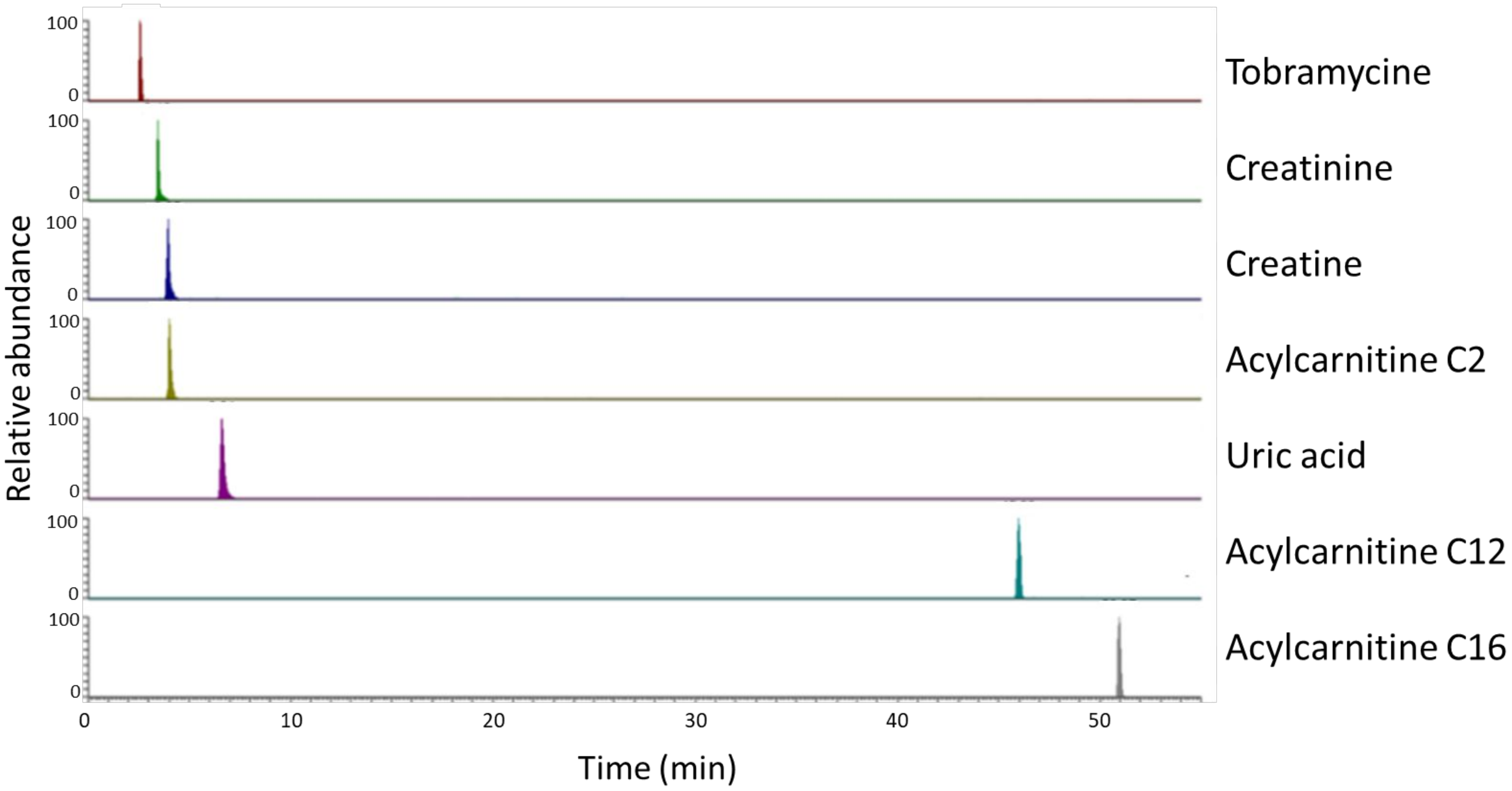

Figure S1. Extracted ion chromatograms of tobramycine, creatinine, creatine, acylcarnitine C2, uric acid, acylcarnitine C12 and acylcarnitine C16 using the Polaris C18-Ether column.

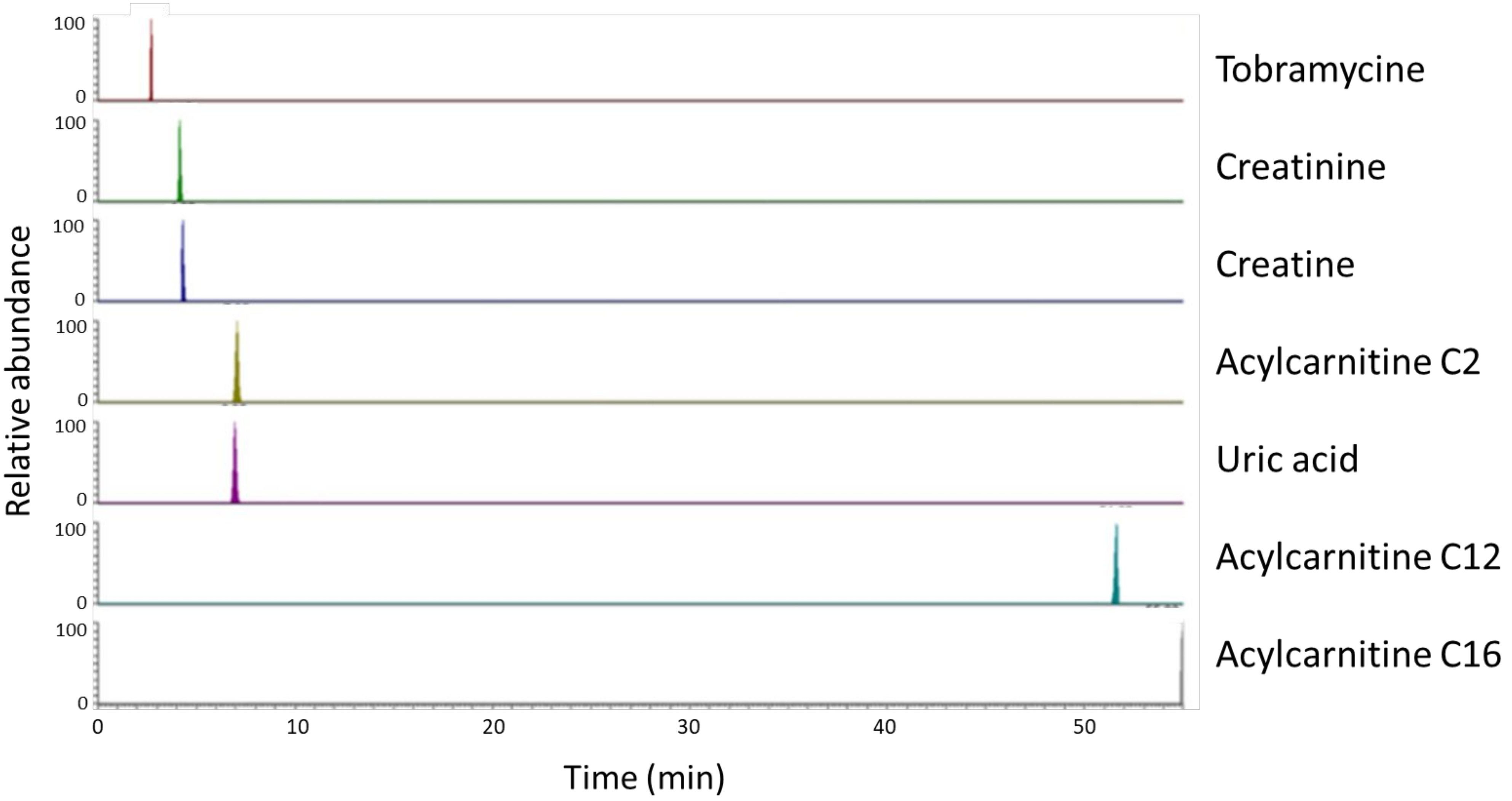

Figure S2. Extracted ion chromatograms of tobramycine, creatinine, creatine, acylcarnitine C2, uric acid, acylcarnitine C12 and acylcarnitine C16 using the ACE C18-PFP column.

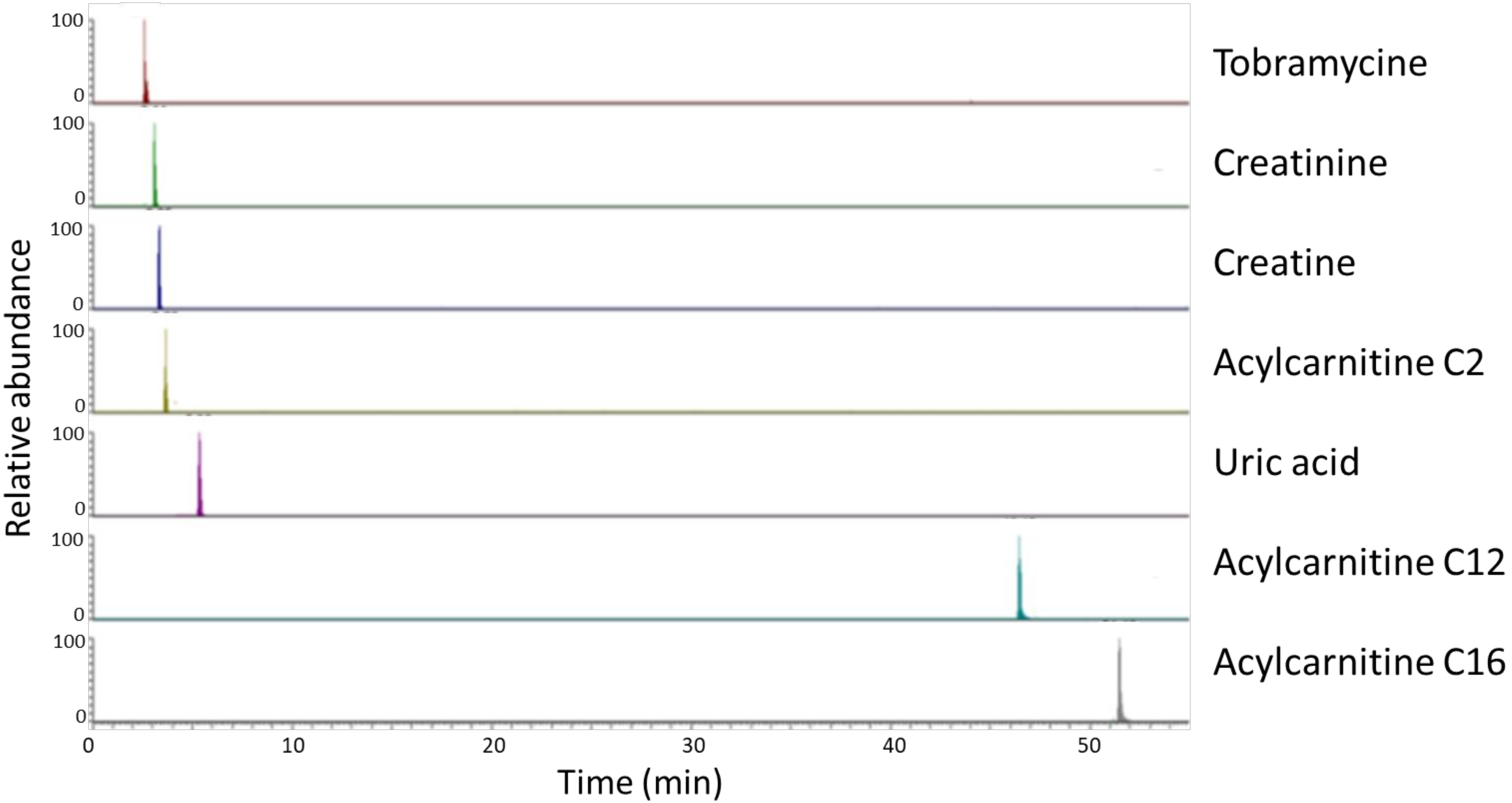

Figure S3. Extracted ion chromatograms of tobramycine, creatinine, creatine, acylcarnitine C2, uric acid, acylcarnitine C12 and acylcarnitine C16 using the Aeris Peptide XB-C18 column.

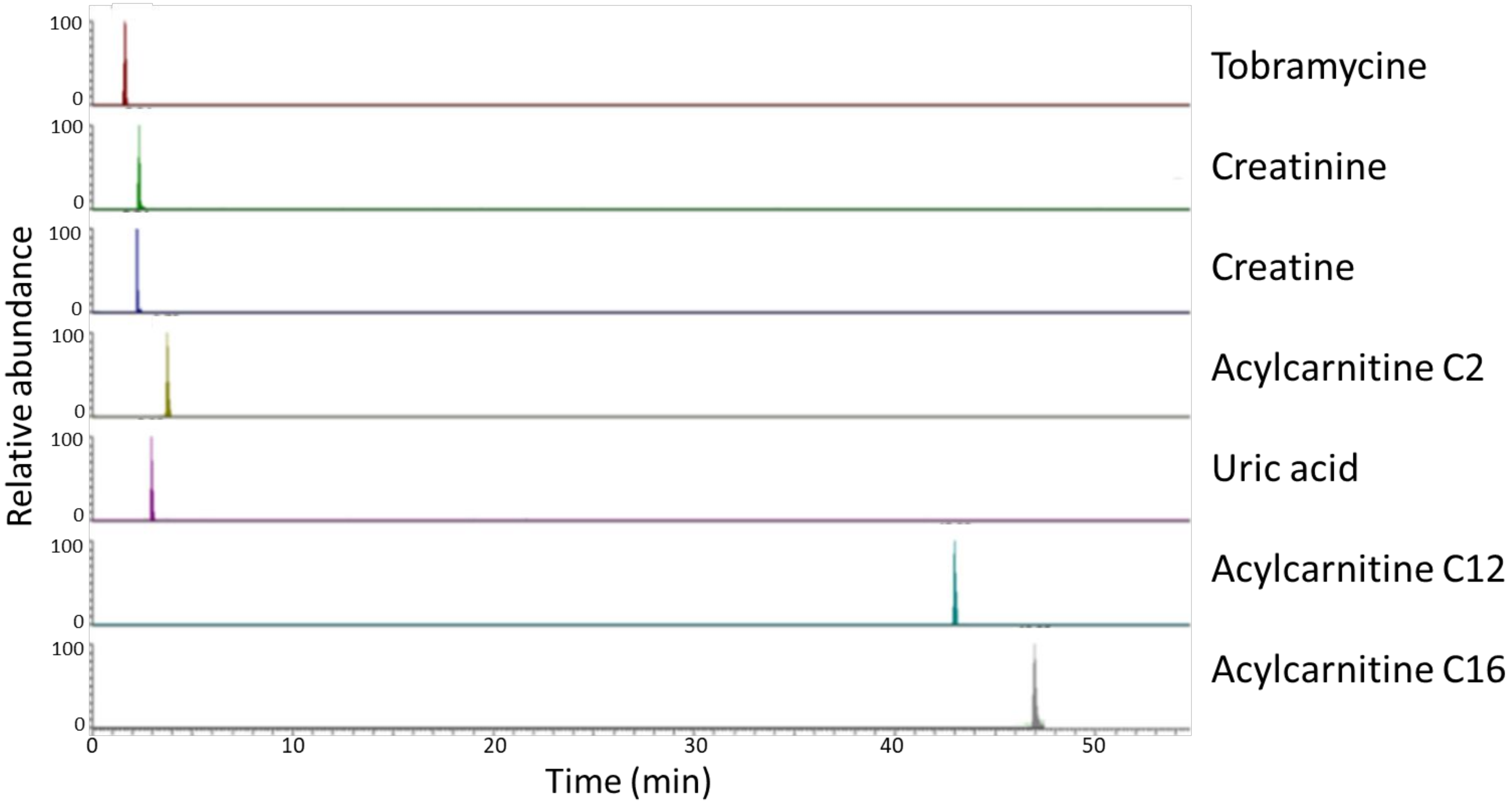

Figure S4. Extracted ion chromatograms of tobramycine, creatinine, creatine, acylcarnitine C2, uric acid, acylcarnitine C12 and acylcarnitine C16 using the Raptor Biphenyl column.
